# A252 LONG-TERM OUTCOMES IN INFLAMMATORY BOWEL DISEASE PATIENTS WITH PRIMARY SCLEROSING CHOLANGITIS: KINGSTON HEALTH SCIENCES CENTER LOCAL EXPERIENCE

**DOI:** 10.1093/jcag/gwac036.252

**Published:** 2023-03-07

**Authors:** M Greenblatt, D Mulder, J Flemming

**Affiliations:** 1 Pediatric Gastroenterology; 2 Adult Gastroenterology, Queen's University, Kingston, Canada

## Abstract

**Background:**

IBD-PSC remains a poorly understood entity due to it’s low incidence and the heterogeneity found within this patient population. Patients with IBD-PSC have 0.3-2.8% lifetime risk of developing hepatocellular carcinoma, up to a 20% lifetime risk of developing Cholangiocarcinoma and a 20-30% lifetime risk of developing Colorectal Cancer (Fung et al. World J Gastroenterol 2019). To better understand long-term outcomes in this population, the use of large population level data can be useful but requires case validation.

**Purpose:**

A large multicenter Canadian retrospective cohort study is ongoing with the goals of identifying an algorithm to identify IBD-PSC patients and compare long-term outcomes of IBD-PSC patients with a matched IBD cohort (Ricciuto et al, ongoing). This abstract describes the local data within Kingston Health Sciences Centre (KHSC) from this Canadian multi-center study and evaluates the use of ICD codes for the identification of IBD-PSC.

**Method:**

This is a single centre retrospective cohort study of patients with IBD-PSC within KHSC from January 1993 - December 2021. Patients with potential IBD-PSC were initially identified locally using ICD codes for IBD and cholangitis. Charts were reviewed and IBD-PSC diagnosis was confirmed using pathology from liver biopsies and imaging. Those confirmed to have IBD-PSC then had data manually extracted from the local health administrative system. Outcomes of interest were divided into patient demographics, disease phenotype at diagnosis of IBD-PSC and therapies. Patients were followed until death, leaving the KHSC network or the end of the follow-up period.

**Result(s):**

Of the 862 patients identified using ICD codes, 16 (2%) were confirmed to have IBD-PSC after chart review. 50% of included patients had other autoimmune diseases. The median age of diagnosis of PSC was 34 years (IQR=22-43) while the median age of diagnosis of IBD was 22 (IQR=15-38). Large duct PSC was found in 88% of patients. Six patients had disease that was exclusively intra-hepatic. Two patients were found to have PSC with auto-immune hepatitis overlap. Mean ALP at diagnosis was 402 (IQR=208-506). Four patients developed cirrhosis, two of which experienced hepatic decompensation. Three patients underwent liver transplant. 81% of patients had ulcerative colitis. At the index lower endoscopy, 33% of patients had Mayo 3 colitis and 75% had involvement of the right colon. During follow up in our study, 81% of patients required systemic steroids and 33% required biologic therapy.

**Conclusion(s):**

ICD codes alone cannot reliably identify patients with IBD-PSC. The characteristics of the patients in our local experience are in keeping with existing literature. Completion of this multi-center study will allow for a greater sample size, a matched control group and a better understanding of the long-term impacts of this disease.

**Please acknowledge all funding agencies by checking the applicable boxes below:**

None

**Disclosure of Interest:**

None Declared

